# Effect of the Replacement of Maize Silage and Soyabean Meal with Mulberry Silage in the Diet of Hu Lambs on Growth Performance, Serum Biochemical Indices, Slaughter Performance, and Meat Quality

**DOI:** 10.3390/ani12223164

**Published:** 2022-11-16

**Authors:** Mingyan Wang, Haoqi Han, Yuan Shang, Liyang Zhang, Yu Zhang, Chuanyou Su, Hongxia Lian, Tong Fu, Tengyun Gao

**Affiliations:** Henan International Joint Laboratory of Nutrition Regulation and Ecological Raising of Domestic Animal, College of Animal Science and Technology, Henan Agricultural University, Zhengzhou 450046, China

**Keywords:** mulberry silage, Hu lambs, growth performance, serum biochemical indices, slaughter performance, meat quality

## Abstract

**Simple Summary:**

The application of maize silage has certain limitations, and the development of new forage resources is of great significance for improving resource utilization and environmental protection. Mulberry leaves are rich in nutritional value and are widely used in animal husbandry. However, excessive antinutritional factors, such as crude fiber, tannins, and phytic acid, in mulberry leaves affect the absorption and the utilization of nutrients by animals. Microbial fermentation technology can significantly reduce the contents of crude fiber, and the antinutritional factors in mulberry leaves minimize nutrient loss and improve the palatability of mulberry leaves. Therefore, this experiment found that the replacement of maize silage and soyabean meal with mulberry silage in the diet of Hu lambs has the potential to enhance muscle nutritional value and to improve mutton flavor without negatively affecting growth performance, serum biochemical indices, slaughter performance, or meat quality.

**Abstract:**

Maize silage has a high demand for fertilizer and water. As an unconventional feed resource, mulberry silage has the potential to replace most maize silage and to alleviate the shortage of roughage in the mutton sheep industry in China. The purpose of this experiment was to study the effect of the replacement of maize silage and soyabean meal with mulberry silage in the diet of Hu lambs on growth performance, serum biochemical indices, slaughter performance, and meat quality. Ninety-six healthy Hu lambs were randomly divided into four groups with six replicates per group and four lambs per replicate. The amounts of 0, 20, 40, and 60% of maize silage were replaced by mulberry silage in each group (denoted as CON, L, M, and H, respectively). The results showed that replacing maize silage with mulberry silage had no significant effect on the growth performance or the slaughter performance of Hu lambs (*p* > 0.05). Feeding Hu lambs with mulberry silage significantly reduced serum glucose (GLU) and the blood urea nitrogen (BUN) content (*p* < 0.05), and it increased the content of ether extract (EE) in the longissimus dorsi muscle (*p* < 0.05). Meanwhile, the percentage of EAA in the M and H groups was significantly lower than that in the CON and L groups (*p* < 0.05). In addition, in the fatty acid profile, the percentage of C16:1 in the M group was significantly increased, while the percentage of C18:0 and C20:0 were significantly decreased (*p* < 0.05). Based on these findings, it was recommended that 20–40% of maize silage be replaced by mulberry silage in the diet of Hu lambs.

## 1. Introduction

In recent years, the expansion of animal husbandry in China has been constrained by the limited land resources available for the production of conventional forages (such as maize silage). China’s maize planting area is thought to be around 1.67 million hectares, which can be harvested 20–25 t/dry matter/ha/year (dry matter is represented as DM) with irrigation needed [1]. Due to the good drought resistance of mulberry, there is almost no need for irrigation during planting, and the yield can reach 20–30 t/DM/ha/year [2]. In actuality, growing maize has a negative impact on the environment. In the United States, maize accounts for about 18% of total ammonia emissions. Large ammonia emissions cause air pollution and a rise in particulate matter (PM), particularly PM2.5, which is a type of particle with a diameter of 2.5 microns or less that is harmful to human respiratory health. At the same time, the need for a nitrogen-based fertilizer in maize production has increased carbon dioxide emissions from the fertilizer industry, which is also bad for combating climate change. By contrast, mulberry, as an internationally recognized bioenergy and biofuel crop [3,4], has a response to supplementation with N, P, and K that is relatively poor. Besides, mulberry leaf flavonoids have been shown in studies to suppress the growth of methanogenic microorganisms, primarily methanogens and protozoans in the rumen, hence reducing CH_4_ production [5,6]. Therefore, replacing maize silage with mulberry silage can not only improve resource utilization but also has the potential to reduce environmental pollution.

Mulberry belongs to Morus, Moraceae, and it is a deciduous tree. It has been planted in China for more than 5000 years, with rich varieties and a wide distribution. Its leaves are mainly used for silkworm rearing, traditional herbal medicine, and animal feed [7]. It also has broad application prospects in the fields of medicine, food, and the chemical industry. Its leaves are considered a high-quality forage resource because of their high protein content, balanced amino acid composition, and richness in other nutrients such as vitamins and mineral elements [8]. At the same time, mulberry leaves are rich in flavonoids, alkaloids, polysaccharides, and other bioactive components, which not only have immune [9], antioxidant [10], lipid-lowering [11], hypoglycemic [12], and other effects but can also enhance animal production performance, improve meat quality, and increase economic benefits [2,13]. However, the crude fiber, tannins, phytic acid, and other antinutritional factors contained in mulberry leaves affect the absorption and utilization of nutrients in animals [14]. Studies have found that the fermentation of silage could significantly reduce the contents of the crude fiber and antinutritional factors in mulberry leaves, minimize nutrient loss, and improve the palatability of mulberry leaves, which is the most commonly used processing method for livestock and poultry feed [15,16]. Microbial fermentation technology can transform the indigestible macromolecules in mulberry leaves into digestible small molecules, such as amino acids, small peptides, and organic acids, and can release their nutrients [17]. In our study, the results showed that, compared with maize silage, the crude protein (CP) and ether extract (EE) content of mulberry silage increased by 9.1% and 2.84%, respectively, and the neutral detergent fiber (NDF) content decreased by 7.08%, which was ideal for ruminant feed materials. In summary, as a new type of protein feed resource, mulberry silage has broad application prospects in animal husbandry.

A large number of studies have confirmed that adding mulberry silage to the diet can improve production performance [18], enhance the antioxidant capacity [19], improve meat quality [13] and increase economic benefits [20]. At the same time, it plays an important role in maintaining ruminant health and changing the rumen bacterial-community structure [21]. However, it has not been fully utilized as feedstuff for ruminants. Hu sheep, with strong adaptability, fast growth, good meat production performance, and a tolerance to roughage, are one of the preferred varieties of mutton sheep unique to China [22]. Therefore, this experiment studied the effects of replacing maize silage and soyabean meal with mulberry silage in the diet of Hu lambs on growth performance, blood biochemical indices, slaughter performance, and meat quality to provide a basis for its application in animal feeding.

## 2. Materials and Methods

From April to June 2021, this study was carried out at Yuemeihe Agriculture and Animal Husbandry Co., Ltd., in Lin Ying County, Henan Province. The Institutional Animal Care and Use Committee (IACUC) of Henan Agriculture University accepted the protocols, and the feeding trial was carried out in compliance with them (Permit Number: 12-1328; Date: 05-2021).

### 2.1. Experimental Design

A total of 96 healthy 3-month-old fattening Hu lambs weighing approximately 27.50 ± 3.03 kg were randomly divided into four groups according to the principle of random grouping, with 6 replicates in each group and 4 lambs in each replicate. Mulberry silage was used to replace 0, 20, 40, and 60% of the maize silage in Hu lamb diets as a total mixed ration (TMR) (denoted as CON, L, M, and H group, respectively). The feed formula was formulated according to the balance of metabolic energy and crude protein (Table 1). Changes in other dietary components were required to ensure that mulberry silage could replace maize silage in equal proportions, meet the 120 g/d growth rate of fattening lambs (NRC, 2007), and maintain the balance between metabolized energy and CP. This eventually resulted in changes to the addition amount of maize and soyabean meal. In CON treatment, 12% (L group), 24% (M group), and 35% (H group) of soyabean allocations were also replaced by mulberry silage. With the increasing proportion of mulberry silage replacing maize silage, the contents of ether extract (EE), CP, and calcium (Ca) increased, while the contents of neutral detergent fiber (NDF) and acid detergent fiber (ADF) decreased. In the design of feed composition, the target CP content was 14.5% (DM). Due to the long test period, the nutritional value of feed ingredients in different batches and producing areas was different, and it eventually led to changes in proteins and other nutrients. However, this did not affect the physiology of Hu lambs, and they could meet their maximum growth needs [23].

The lamb house was a well-lit, partially closed, portal-framed structure that was kept at a temperature between 18 and 28 ℃. An acclimation period of 15 days and a formal trial of 60 days made up the 75-day duration of the experiment. The Hu lambs were fed TMR feed ad libitum twice daily at 07:00 and 17:00 during the breeding period. Daily feed residues were weighed, and the ration supply was changed to ensure that 5% of the feed was left over every day. There was a separate drinking bowl in each pen, allowing for unlimited use.

### 2.2. Feeding and Management of Experimental Animals

Before the start of the experiment, the pens were regularly sterilized with 3% Lysol twice a week, and all the experimental lambs were dewormed and earmarked according to normal immune and management procedures. The health of the flock was checked daily, lambs with fever and cough were recorded and treated, and no test animals died during the whole test period.

### 2.3. Experimental Materials

Mulberry silage was provided by Yuemeihe Agriculture and Animal Husbandry Development Co., Ltd., Linying County, China, for use in this experiment. Once the mulberry trees reached 1.5 m in height, they could be harvested using the silage harvester (Claas jaguar 800, Wister, Germany), leaving stubble heights between 15 and 20 cm. Because mulberry trees regrow three to four days after harvesting, they could be harvested three to four times a year. Freshly harvested mulberries were chopped to 1 to 2 cm, and then 1 g each of purchased mature strains of Chr Hansen *lactococcus lactis* and *lactobacillus brucei* powder (lactic acid bacteria > 1.3 × 10^11^ CFU/g) were added to each ton of mulberry. After mixing evenly, the silage was wrapped by the automatic silage packer (Qufu Xinlian Heavy Industry XL-5552, Shandong, China) and was fermented in a cool and dry place.

The maize silage used in this experiment was provided by Yuemeihe Agriculture and Animal Husbandry Development Co., Ltd., Linying County, China. The harvest period of maize silage was milk-ripe stage, the stubble height was approximately 10 cm, and the whole plant was cut to 2~5 cm. When the water content was approximately 70%, the automatic silage packer was used to coat the silage which was placed in a cool and dry place for fermentation. After fermentation, dry matter (DM), ash, EE, Ca, and phosphorus (P) were determined by AOAC method 920.39, AOAC method 942.05, AOAC method 920.85, AOAC method 985.35, and AOAC method 986.24, respectively; CP was measured using the method described by Kjeltec 2300 analyzer (FOSS Analytical AB, Hoganas, Sweden); and NDF and ADF were tested using the methods described by Van Soest [24] and Ankom Fiber Analyzer (Ankom Technology, Fairport, NY, USA). The detected nutritional components of mulberry silage and maize silage are shown in Table 2.

### 2.4. Growth Data Collection and Analysis

All lambs were weighed for two consecutive days before morning feeding each month using the Guangdong Senssun Weighing Apparatus in China (error + 0.01 kg). The feeding and residual amounts were recorded in the unit of experiment repetition, and the dry matter intake (DMI), ADG, and feed conversion ratio (DM intake/live weight gain) were calculated by lamb weight, feeding amount, and residual amount.

### 2.5. Blood Collection and Biochemical Analysis

A disposable vacuum blood sample needle was used to collect jugular vein blood from 12 randomly selected lambs in each group before morning feeding on the last day of the formal trial. Two 15 mL centrifuge tubes, one with and one without EDTA anticoagulant, were used to collect blood samples. The blood samples without an anticoagulant were left at room temperature for 30 min, then they were centrifuged for 15 min at a speed of 3000 rpm/min. The supernatant was then collected and stored at −20 °C until used. The Beckman Kurt AU5800 automatic biochemical analyzer was used to detect and analyze the blood biochemical indices in accordance with the manufacturer’s instructions (Jian Cheng Bioengineering Institute, Nanjing, China) [22].

### 2.6. Carcass Data Collection and Analysis

At the end of the experiment, 12 lambs from each group were selected randomly and slaughtered. In accordance with Regulation (EC) No. 1099/2009, the Hu lambs were transported to a nearby slaughterhouse and then slaughtered after high voltage shock. Before that, they were fasted for 24 h and deprived of water for 2 h [25]. The carcass weight, live weight before slaughter, carcass meat rate, sheepskin weight, bone weight, liver weight, lung weight, rumen weight, and omental weight were measured. Dressing percentage = (carcass weight/live weight before slaughter) × 100%; carcass meat rate = (meat weight/carcass weight) × 100%.

### 2.7. Longissimus Dorsi Muscle Sampling

After slaughter, samples of longissimus dorsi muscle (approximately 300 g) between the 12th and 13th ribs of the left lung of the carcass were collected. The pH value of the longissimus dorsi muscles was measured with a handheld pH meter (Russell CD700, Russell pH Limited, München, Germany) at 45 min (pH_45min_) and 24 h (pH_24h_). The pH measurement of each muscle was repeated five times and averaged, and the mean was statistically analyzed as appropriate. A portion of each sample was dispensed into two 2 mL centrifuge tubes and stored in liquid nitrogen, and the rest was stored in a 4 ℃ refrigerator for subsequent use.

### 2.8. Muscle Chemical Composition Analysis

The longissimus dorsi muscle samples were thawed, and all the visible external fat on their surface was removed. Then the samples were cut into small pieces, were fully mixed, and were randomly sampled for subsequent operation. After drying, the samples were ground using a grinder for subsequent determination of DM, EE, ash, and CP contents in reference to the method of Guzman et al. [26].

### 2.9. Amino Acid Composition of Muscle Analysis

Amino acids in muscle samples were determined by ion exchange AA analyzer (Ninhydrin postcolumn derivatization ion exchange chromatography, Germany) according to GB 5009.124-2016 National Food Safety Standards. About 50 mg of each dried sample was hydrolyzed in 10 mL of 6 mol/L HCl at 110 °C for 24 h. The pH of each hydrolysate was adjusted to about 9, and then the volume of each was fixed to 50 mL. Each of the above hydrolysis solutions were added with derivative agents A and B for precolumn derivatization and then were filtered with 0.45 μm of organic membrane. The filtrates were analyzed by ion exchange AA analyzer, and the results were expressed as percent of total amino acids [22,27].

### 2.10. Fatty Acid Composition of Muscle Analysis

After drying and crushing the muscle samples, 0.5 g of each was weighed and placed in a 15 mL plug test tube. 4 mL isooctane was added to each, was shaken in a vortex mixer for 30 s, and then was shaken overnight in a 37 °C constant temperature shaker. Subsequently, 4 mL of 2 mol/L potassium hydroxide-methanol solution was added to each and was vortexed for 30 s to rapidly methylate the samples. Next, after standing for 30 min for stratification, about 1 g of sodium bisulfate was added to each test tube and was shaken violently to neutralize the remaining potassium hydroxide. After standing until a clear solution appeared in the upper layer of the test tubes, the clear solutions in the upper layers were filtered through a 0.45 μm microporous membrane and were placed in a SCION 456-GC gas chromatograph (Bruker Daltonics) to analyze fatty acid composition [13]. Strong polar polyethylene glycol was used as the stationary phase in the chromatographic column (column length was 30 m, inner diameter was 0.250 mm, film thickness was 0.25 m). The temperature was raised in the following order: 50 °C for one minute, 25 °C/min to 220 °C, then 3 °C/min to 230 °C for 18 min. The temperatures of the injector and detector were 220 °C and 280 °C, respectively. The gas flow rate was 30 mL/min, and the carrier employed was nitrogen. Finally, fatty acids were determined by contrasting the peak’s retention time with established standards (Sigma, St. Louis, MO, USA).

### 2.11. Statistical Analysis

In this study, SPSS 25.0 was used for one-way ANOVA (with linear and quadratic contrasts) and Duncan’s multiple comparison. Mathematical model used for analysis was *y_ij_* = *u_i_* + *δ_i_* + *ε_ij_*, where *y_ij_* is the dependent variable, *u_i_* is the overall mean, *δ_i_* is the fixed effect of mulberry silage residues (*i* = 0, 20, 40, 60% of maize silage being replaced by mulberry silage), and *ε_ij_* is the random residual error. Data were expressed as the mean ± standard error (SEM), and results with *p* < 0.05 indicated a significant difference.

## 3. Results

### 3.1. Growth Performance

Table 3 shows that there were no significant differences in initial body weight (IBW) and final body weight (FBW) among the groups (*p* > 0.05). At the same time, the replacement of maize silage mulberry silage in the diet of Hu lambs had no significant effect on dry matter intake (DMI) (*p* > 0.05). Meanwhile, compared with CON group, the average daily gain (ADG) of L and M groups tended to increase, feed conversion ratio (DM intake/live weight gain) also improved, but there were all no significant difference (*p* > 0.05).

### 3.2. Serum Biochemical Indices

As shown in Table 4, compared with the CON group, the content of glucose (GLU) decreased linearly (*p* < 0.05), and the content of blood urea nitrogen (BUN) changed in a quadratic curve (*p* < 0.05) with the increase in the proportion of mulberry silage. Among them, the BUN in the M group presented significantly lower values with regard to the H group, but there was no statistical significance among the CON, L, and M groups. However, there was no significant difference in other blood indices among all the groups (*p* > 0.05).

### 3.3. Slaughter Performance

Table 5 shows that the replacement of maize silage with mulberry silage in the diet had no significant effect on the dressing percentage or the carcass meat rate of Hu lambs (*p* > 0.05). At the same time, there were no significant differences in sheepskin weight, bone weight, lung weight, liver weight, rumen weight, and greater omentum weight among all groups (*p* > 0.05).

### 3.4. Muscle Chemical Composition

As shown in Table 6, feeding mulberry silage had no significant effect on the contents of the DM, CP, and ash in the muscle of Hu lambs (*p* > 0.05), but the content of the EE in the M group was significantly higher than that in the CON group (*p* < 0.05). At the same time, there was no significant difference in the pH_45min_ and PH_24h_ values of the longissimus dorsi muscles among all the groups (*p* > 0.05), which was within the normal range.

### 3.5. Amino Acid Profiles

From Table 7, with the increase of the proportion of mulberry silage replacing maize silage, the percentage of proline (Pro), tyrosine (Tyr), valine (Val), isoleucine (Ile), leucine (Leu), and phenylalanine (Phe) was linearly changed (*p* < 0.05); aspartic acid (Asp), glycine (Gly), and cysteine (Cys) showed quadratic changes (*p* < 0.05). Among them, compared with the CON group, there was no statistical difference in the L group except that Tyr was significantly increased. In addition, compared with the CON, the percentage of Asp and Pro in the M and H groups was significantly increased, while the percentage of Gly, Val, and Phe in the H group was significantly decreased. In the amino acid profiles, Leu (10.44–11.00%) and proline (Pro) (8.12–8.47%) accounted for the largest proportions, followed by lysine (Lys) (7.75–8.27%) and Arg (7.69–8.18%). Essential amino acids (EAA) accounted for 43.56–46.39% of the total amino acids, and the percentage of EAA in the M and H groups was significantly lower than that in the CON and L groups (*p* < 0.05).

### 3.6. Fatty Acid Profiles

Table 8 reported the composition of fatty acids (% of total fatty acids) in the longissimus dorsi muscles. In the saturated fatty acid profile, the percentage of C14:0, C16:0, C18:0, and C20:0 showed quadratic changes (*p* < 0.05). Compared with the CON group, the percentage of C16:0 in the M group was significantly increased, and the percentage of C18:0 and C20:0 was significantly decreased. Meanwhile, the percentage of C14:0 and C16:1 in the M group was significantly higher than that in the CON and H groups (*p* < 0.05). We also observed that C18:1 accounted for the largest proportion in all fatty acid profiles, and the M group had the highest MUFA value and the lowest PUFA value. However, these were all of no significant difference (*p* > 0.05).

## 4. Discussion

Compared with other commonly used feed, mulberry silage has a higher digestibility and a better palatability. This study found that the replacement of maize silage with mulberry silage in the diet had no negative impact on the growth performance of Hu lambs, which was consistent with Wu et al. [20]. As an important fiber index, NDF could increase the satiety of animals and was negatively correlated with feed intake [28,29]. Therefore, it was speculated that a small increase in the DMI may be related to the NDF content in the diet.

Mulberry leaves contain a variety of bioactive components, among which 1-deoxynojirimycin (1-DNJ), the main active alkaloid substance, is a glycosidase inhibitor that can significantly improve the glucose utilization efficiency and reduce blood glucose levels [30]. GLU is the main energy source of the body, and its content changes show a dynamic balance, reflecting the synthesis and metabolism of carbohydrates in the body and affecting muscle lipid metabolism and protein conversion, thereby improving meat quality [31]. The study found that the GLU concentration decreased significantly after feeding with mulberry silage, and the effect was more significant with the increase of the replacement rate, suggesting that this may be related to 1-DNJ. LDH is an important enzyme involved in anaerobic glycolysis and gluconeogenesis that can catalyze the redox reaction between pyruvate, L-lactic acid, and the related α-ketoacid. The content of LDH in serum increased slightly after feeding with mulberry silage, which may have been affected by glucose metabolism, but the difference between the groups was not significant. Serum transaminase is an important indicator for detecting the physiological functions of the liver and heart [32]. The changes in the diet did not affect the contents of AST and ALT in serum, indicating that mulberry silage had no adverse effects on the livers and hearts of Hu lambs. In serum, TG, TCHO, HDL-C, and LDL-C are important components of blood lipids, and their levels reflect the lipid metabolism in animals [33]. Serum TP, GLB, GLO, and BUN have important nutritional functions, and they reflect the health status of animals. BUN is the final product of protein hydrolysis and amino acid metabolism, and its content is negatively correlated with body nitrogen deposition and protein utilization [8,34]. In this experiment, the TP, GLB, and GLO in each group were in a stable state, the BUN in the M group was significantly lower than that in the CON group, which was consistent with the results of Sun et al. [35]. Specifically, the lower BUN content was caused by the fact that the majority of the ammonia created during the breakdown of the crude protein in the rumen was used to create microbial protein and that only a tiny amount of ammonia was transferred into the liver by the blood to create urea.

An important gauge of the economic worth of meat livestock and poultry is slaughter performance [36,37,38]. The study found that the effect of including mulberry in Hu lamb feed on the overall acceptability of slaughter performance was in the terms of the dressing percentage and the carcass meat rate, which was consistent with the findings of Ouyang et al. [39]. Similar slaughter performances indicated that the use of mulberry silage had no detrimental effect on Hu lambs. According to research, adding dietary fiber to juvenile ruminant concentrate may have an impact on rumen development and body weight, and the degree to which this impact varies depends on the source and content of the fiber [40]. In this study, the replacement of maize silage with mulberry silage in the diet did not affect the rumen weight of Hu lambs, indicating that the dietary changes had no effect on the rumen weight of Hu lambs.

Muscle contains moisture, protein, fat, ash, and other physical and chemical indices. A high EE content in mutton will lead to excessive cholesterol consumption and an increase in cardiovascular illnesses, which is contrary to the idea of public consumption, while a low EE content will impair the flavor. Particularly, the low fat content of lambs will result in less deposition of various flavor precursors particular to lambs in the body as well as a light odor [41]. According to studies, dietary nutrition directly influences muscle fatty acid composition and fat deposition, which in turn influences the flavor, tenderness, and juiciness of the meat [42]. As a result, by altering the dietary energy density, the intramuscular and intermuscular fat contents can be balanced [8]. Mulberry silage contains a high fat content as well as secondary compounds that may improve animal health and meat quality, making it a great potential as an excellent feed resource [43,44,45]. In this study, it was found that the replacement of maize silage with mulberry silage in diet significantly increased the EE content in longissimus dorsi muscles of Hu lambs. Studies have shown that the flavonoids rich in mulberry leaves have a regulatory effect on animal fat deposition and lipid metabolism [46], which might be the reason for the increase in the EE content after maize silage was replaced by mulberry silage in this experiment. Studies have shown that the final pH value of mutton after slaughter should be between 5.6 and 6.4 [47]. In particular, Della Malva et al. [48] suggested that 24 h after slaughter, the muscle pH values of lambs were between 5.5 and 5.8, which belongs to the normal level of pH_24h_ values. In our study, although there was no significant difference in the pH values of the longissimus dorsi muscles after feeding with mulberry silage in each group, they were within the normal range, which was consistent with the findings of Majdoube-mathlouthi et al. [49]. It is speculated that this may be caused by the antioxidant properties of the flavonoids, polysaccharides, alkaloids, and other bioactive components found in mulberry leaves, which can protect lipids from oxidation, thereby inhibiting mutton rancidity, delaying the pH decline rate, and improving meat quality. Collectively, adding mulberry silage instead of maize silage in Hu lamb diets can effectively improve the nutritional value of the meat.

Its nutritional value and flavor are significantly influenced by the composition and amount of amino acids and saturated fatty acids in the muscle. Studies have shown that the amount of EAA in the muscle plays a significant role in determining the quality of the meat, and the protein requirements for lamb growth may also be determined from the composition of EAA [50]. In this study, EAA accounted for 43.56–46.39% of the total amino acid content in each group, which was in line with the World Health Organization requirements for amino acid intake in human diets [51]. At the same time, this study also found that the amino acids in the muscle of Hu lambs were mainly Leu, Pro, Lys, and Arg, which accounted for a large proportion of the amino acid composition, and they were also the main components of muscle EAA, which was consistent with the results of Wu et al. [52]. Saturated fats are mainly derived from baking products, red meat, and high-fat dairy products, which, together, contribute to 51% of the saturated fatty acid (SFA) intake in adolescents [53]. The intake of SFAs is a risk factor for inducing cardiovascular diseases. Therefore, public health policy states that humans should reduce their intake of SFAs [54]. However, Napolitano et al. [55] reported that a higher C18:0 content in the diet, which is not easily digested and easily desaturated to oleic acid(C18:1), may not raise LDL cholesterol. Thus, not all SFAs have the potential to induce cardiovascular diseases. A high unsaturated fatty acid (UFA) content in mutton is an important health characteristic, but an excessive UFA level will affect the oxidative stability of mutton and produce a negative odor [56]. The dietary changes did not affect the UFA content in the muscles, indicating that mulberry silage had no negative effect on the healthiness of Hu lambs. A strong inverse relationship between the MUFA concentration and shear force has been reported [57]. At the same time, Li et al. [58] and Zhang et al. [59] also said that dietary changes lead to a lower IMF content and may reduce C16:1 and C18:1 de novo synthesis by reducing the concentration of saturated substrates, resulting in the increased hardness and shear force of the meat. In our study, MUFA accounted for 40.37–47.10% of the total fatty acid content, of which the main MUFA was C18:1, and the dietary changes significantly increased the percentage of C16:1. This confirmed that feeding with mulberry silage has the potential to improve meat quality, but further studies are needed.

## 5. Conclusions

Based on our results, we concluded that replacing maize silage with mulberry silage in the diet of Hu lambs had no negative effects on growth performance, serum biochemical indices, and slaughter performance. Mulberry silage partially replaced maize silage to reduce the blood GLU and BUN content of Hu lambs. In addition, mulberry silage also increased the muscle EE content, but too high of a replacement ratio would have a negative effect on EAA. Therefore, under the conditions of this experiment, it was best to replace 20-40% of maize silage with mulberry silage.

## Figures and Tables

**Table 1:** Ingredients and chemical compositions of experimental diets.

Items	Group ^3^			
	0	L	M	H
Ingredients (g/kg DM)				
Maize	200	220	240	260
Wheat bran	100	100	100	100
Soyabean meal	170	150	130	110
Maize silage	250	200	150	100
Mulberry silage	0	50	100	150
Peanut vines	250	250	250	250
Premix ^1^	20	20	20	20
Bicarbonate of soda	10	10	10	10
Total	1000	1000	1000	1000
Nutrient concentrations ^2^ (g/kg DM, except where otherwise stated)				
Dry matter (g/kg, fresh weight)	445.1	444.1	443.2	441.8
Digestible energy (DE), MJ/kg	12.3	12.0	12.0	11.8
Crude protein (CP)	135.6	137.1	142.8	149.3
Ether extract (EE)	27.7	29.5	28.9	30.8
Neutral detergent fiber (NDF)	394.7	378.2	370.3	367.8
Acid detergent fiber (ADF)	250.8	241.0	238.3	225.8
Ash	122.8	116.5	117.4	109.4
Calcium (Ca)	13.3	13.2	15.2	16.5
Phosphorus (P)	2.2	2.2	2.2	2.5

^1^ Per kilogram of the concentrate, the premix offered the following: vitamin A 1.0 × 10^4^ IU, vitamin D3 2000 IU, vitamin E 125 IU, niacin 250 mg, pantothenic acid 75 mg, biotin b 5 mg, Cu 20 mg, Fe 68 mg, Mn 56 mg, Zn 50 mg, I 1.05 mg, Se 0.2 mg, and Co 0.75 mg. ^2^ Nutrient levels were measured values. ^3^ CON, L, M, and H represent the replacement of 0, 20, 40, and 60% of maize silage with mulberry silage, respectively.

**Table 2:** Nutrient composition of mulberry silage and maize silage (g/kg DM, except where otherwise stated).

Items	DM (g/kg, Fresh Weight)	EE	CP	Ash	NDF	ADF	Ca	P	GE ^1^ (MJ/kg)
Mulberry silage	247.6	73.8	169.0	87.2	429.9	284.2	11.7	4.0	17.05
Maize silage	308.1	45.4	78.0	80.1	500.7	298.7	6.2	1.1	16.51

^1^ GE and the other nutrient levels were measured.

**Table 3:** Effects on growth performance of Hu lambs by substituting 0 (CON group), 20 (L group), 40 (M group), and 60% (H group) of maize silage with mulberry silage.

Items	Group ^1^				SEM	*p-*Value	
	CON	L	M	H		Linear	Quadratic
Initial body weight (IBW), kg	27.38	27.94	27.05	28.01	0.36	0.757	0.783
Final body weight (FBW), kg	41.21	42.28	41.45	41.67	0.41	0.885	0.616
Average daily gain (ADG), g/d	231	239	240	228	3.52	0.810	0.147
Dry matter intake (DMI), kg/d	1.168	1.170	1.175	1.178	0.01	0.080	0.098
Feed conversion ratio (DM intake/live weight gain)	5.13	4.98	4.90	5.20	0.07	0.839	0.124

^1^ CON, L, M, and H represent the replacement of 0, 20, 40, and 60% of maize silage with mulberry silage, respectively. SEM represents standard error of mean, and *n* = 6.

**Table 4:** Effects on serum biochemical indices of Hu lambs by substituting 0 (CON group), 20 (L group), 40 (M group), and 60% (H group) of maize silage with mulberry silage.

Item	Group ^1^				SEM	*p*-Value	
	CON	L	M	H		Linear	Quadratic
Lactic dehydrogenase (LDH), U/L	501.33	551.50	578.00	558.50	17.50	0.225	0.336
Aspartate aminotransferase (AST), U/L	98.67	107.17	104.50	113.67	4.45	0.317	0.972
Alanine aminotransferase (ALT), U/L	15.17	16.83	18.83	18.67	0.99	0.182	0.655
AST/ALT	7.73	6.38	5.73	6.77	0.63	0.555	0.378
Blood urea nitrogen (BUN), mmol/L	7.56 ^bc^	7.92 ^bc^	6.49 ^c^	9.20 ^a^	0.28	0.072	0.010
Glucose (GLU), mmol/L	4.84 ^a^	4.38 ^ab^	4.01 ^b^	4.00 ^b^	0.12	0.004	0.283
Total cholesterol (TCHO), mmol/L	1.47	1.48	1.44	1.51	0.03	0.835	0.707
Triglyceride (TG), mmol/L	0.30	0.26	0.28	0.26	0.01	0.441	0.695
High-density lipoprotein (HDL-C), mmol/L	0.73	0.80	0.77	0.80	0.02	0.419	0.660
Low-density lipoprotein (LDL-C), mmol/L	0.55	0.53	0.52	0.54	0.02	0.844	0.533
Total protein (TP), g/L	71.80	72.10	69.83	74.25	0.94	0.554	0.288
Albumin (ALB), g/L	34.38	36.43	35.00	37.50	0.60	0.143	0.848
Globulin (GLO), g/L	37.42	35.67	34.83	36.75	1.10	0.787	0.438
ALB/GLO (A/G)	0.93	1.03	1.03	1.03	0.04	0.401	0.530

^1^ CON, L, M, and H represent the replacement of 0, 20, 40, and 60% of maize silage with mulberry silage, respectively. In the same row, mean values with different superscripts indicate significant differences (*p* < 0.05). SEM represents standard error of mean, and *n* = 6.

**Table 5:** Effects on slaughter performance of Hu lambs by substituting 0 (CON group), 20 (L group), 40 (M group), and 60% (H group) of maize silage with mulberry silage.

Item	Group ^1^				SEM	*p*-Value	
	CON	L	M	H		Linear	Quadratic
Dressing percentage, %	51.62	51.83	52.15	51.91	0.00	0.785	0.819
Carcass meat rate, %	79.66	79.38	80.66	79.30	0.01	0.968	0.643
Sheepskin weight, kg	3.02	2.80	2.93	3.00	0.05	0.902	0.195
Bone weight, kg	3.79	3.53	3.59	3.49	0.10	0.361	0.709
Liver weight, kg	0.88	0.79	0.87	0.90	0.02	0.430	0.172
Lung weight, kg	0.65	0.60	0.56	0.59	0.03	0.432	0.451
Rumen weight, kg	1.36	1.27	1.33	1.29	0.02	0.457	0.556
Greater omentum weight, kg	0.25	0.42	0.39	0.40	0.03	0.105	0.159

^1^ CON, L, M, and H represent the replacement of 0, 20, 40, and 60% of maize silage with mulberry silage, respectively. SEM represents standard error of mean, and *n* = 6.

**Table 6:** Effects on muscle chemical composition of Hu lambs by substituting 0 (CON group), 20 (L group), 40 (M group), and 60% (H group) of maize silage with mulberry silage.

Item	Group ^1^				SEM	*p-*Value	
	CON	L	M	H		Linear	Quadratic
Dry matter (DM), %	70.1	69.1	69.1	68.2	0.00	0.662	0.986
Crude protein (CP), g/kg	725.9	716.7	721.9	723.3	0.42	0.953	0.571
Ether extract (EE), g/kg	87.1 ^b^	102.6 ^ab^	116.3 ^a^	90.2 ^ab^	0.01	0.603	0.043
Ash, g/kg	49.0	48.7	48.8	47.7	0.00	0.694	0.860
pH_45min_	6.15	6.49	6.14	6.23	0.08	0.881	0.417
pH_24h_	5.71	5.65	5.61	5.68	0.02	0.289	0.651

^1^ CON, L, M, and H represent the replacement of 0, 20, 40, and 60% of maize silage with mulberry silage, respectively. In the same row, mean values with different superscripts indicate significant differences (*p* < 0.05). SEM represents standard error of mean, and *n* = 6.

**Table 7:** Effects on amino acid profiles in longissimus dorsi muscles of Hu lambs by substituting 0 (CON group), 20 (L group), 40 (M group), and 60% (H group) of maize silage with mulberry silage (% of total amino acids).

Item	Group ^1^				SEM	*p-*Value	
	CON	L	M	H		Linear	Quadratic
Aspartic acid (Asp)	0.22 ^bc^	0.21^c^	0.23 ^b^	0.23 ^a^	0.02	0.000	0.001
Glutamic acid (Glu)	6.45	6.40	6.27	6.29	0.64	0.111	0.775
Serine (Ser)	6.78	6.53	6.17	6.26	0.51	0.461	0.137
Glycine (Gly)	7.20 ^b^	7.09 ^b^	7.06 ^b^	7.12 ^a^	0.41	0.000	0.045
Histidine (His)	6.39	6.13	5.89	5.96	0.42	0.233	0.086
Arginine (Arg)	8.18	7.87	7.69	7.71	0.57	0.151	0.133
Threonine (Thr)	4.69	4.33	4.00	4.06	0.46	0.677	0.141
Alanine (Ala)	7.86	7.61	7.33	7.37	0.49	0.168	0.108
Proline (Pro)	8.27 ^b^	8.12 ^b^	8.47 ^a^	8.33 ^a^	0.52	0.000	0.089
Tyrosine (Tyr)	4.15 ^b^	5.14 ^a^	5.00 ^a^	5.04 ^a^	0.60	0.000	0.156
Valine (Val)	3.33 ^ab^	3.19 ^b^	3.20a ^b^	3.21 ^a^	0.21	0.015	0.056
Methionine (Met)	7.91	7.68	7.51	7.46	0.43	0.056	0.095
Cysteine (Cys)	2.13 ^ab^	1.98 ^b^	1.92 ^b^	2.14 ^a^	0.25	0.083	0.023
Isoleucine (Ile)	6.10 ^ab^	5.96 ^b^	5.87 ^ab^	5.77 ^a^	0.31	0.028	0.127
Leucine (Leu)	11.00 ^ab^	10.68 ^b^	10.60 ^ab^	10.44 ^a^	0.56	0.016	0.089
Phenylalanine (Phe)	5.09 ^b^	4.96 ^b^	4.96 ^ab^	4.86 ^a^	0.26	0.008	0.114
Lysine (Lys)	8.27	8.09	7.83	7.75	0.60	0.254	0.310
EAA ^2^	46.39 ^a^	44.88 ^ab^	43.98 ^b^	43.56 ^b^	0.00	0.00	0.290

^1^ CON, L, M, and H represent the replacement of 0, 20, 40, and 60% of maize silage with mulberry silage, respectively. ^2^ EAA (essential amino acids) = Thr + Val + Met + Ile + Leu + Phe + Lys. In the same row, mean values with different superscripts indicate significant differences (*p* < 0.05). SEM represents standard error of mean, and *n* = 6.

**Table 8:** Effects on the fatty acid profiles in the longissimus dorsi muscles of Hu lambs by substituting 0, 20, 40, and 60% of maize silage with mulberry silage (% of total fatty acids).

Item	Group ^1^				SEM	*p*-Value	
	CON	L	M	H		Linear	Quadratic
SFA ^2^	41.16	42.94	42.11	40.55	0.00	0.537	0.091
C10:0	0.07	0.06	0.07	0.09	0.01	0.398	0.384
C12:0	0.07	0.06	0.06	0.04	0.00	0.400	0.326
C14:0	0.96 ^b^	1.46 ^a^	1.55 ^a^	1.01 ^b^	0.08	0.637	0.000
C15:0	0.19	0.27	0.20	0.29	0.03	0.418	0.921
C16:0	17.83 ^bc^	20.68 ^ab^	21.52 ^a^	18.05 ^bc^	0.49	0.667	0.001
C17:0	0.90	0.88	0.85	0.89	0.02	0.745	0.357
C18:0	20.51 ^a^	19.09 ^ab^	17.49 ^b^	19.61 ^ab^	0.45	0.260	0.045
C20:0	0.15 ^a^	0.13 ^ab^	0.11 ^b^	0.14 ^ab^	0.00	0.282	0.009
C21:0	0.39	0.26	0.20	0.34	0.03	0.462	0.048
C22:0,	0.08	0.05	0.04	0.08	0.01	0.911	0.140
MUFA ^3^	40.31	43.80	47.10	42.98	0.01	0.273	0.105
C14:1	0.02	0.08	0.06	0.05	0.01	0.463	0.081
C16:1	1.58 ^b^	1.74 ^ab^	1.92 ^a^	1.59 ^b^	0.05	0.612	0.011
C18:1	38.51	41.81	44.95	41.14	1.13	0.275	0.122
C20:1	0.20	0.17	0.17	0.19	0.01	0.769	0.230
PUFA ^4^	18.54	13.26	10.79	16.48	0.01	0.481	0.056
C18:2	12.04	8.93	7.50	10.94	0.87	0.539	0.068
C18:3n-6	0.07	0.06	0.05	0.06	0.00	0.187	0.122
C18:3n-3	0.26	0.24	0.22	0.37	0.03	0.186	0.095
C20:2	0.08	0.07	0.06	0.08	0.01	0.884	0.246
C20:3n-3	6.09	3.97	2.97	5.03	0.51	0.342	0.042
UFA ^5^	58.84	57.06	57.89	59.45	0.48	0.537	0.050
PUFA/SFA	0.46	0.32	0.26	0.41	0.04	0.273	0.105

^1^ CON, L, M, and H represent the replacement of 0, 20, 40, and 60% of maize silage with mulberry silage, respectively. ^2^ SFA (saturated fatty acid) = C10:0 + C12:0 + C14:0 + C15:0 + C16:0 + C17:0 + C18:0 + C20:0 + C21:0 + C22:0; ^3^ MUFA (monounsaturated fatty acids) = C14:1 + C16:1 + C18:1 + C20:1; ^4^ PUFA (polyunsaturated fatty acid) = C18:2 + C18:3n-6 + C18:3n-3 + C20:2 + C20:3n-3; and ^5^ UFA (unsaturated fatty acids) = C14:1 + C16:1 + C18:1 + C18:2 + C18:3n-6 + C18:3n-3 + C20:1 + C20:2 + C20:3n-3. In the same row, mean values with different superscripts indicate significant differences (*p* < 0.05). SEM represents standard error of mean, and *n* = 6.

## Data Availability

The data used in this study are available from the corresponding authors on request.
